# Strategies, Facilitators & Barriers in Recruiting Hispanic/Latino Older Adults in NYC Community Organizations

**DOI:** 10.1093/geroni/igaf122.057

**Published:** 2025-12-31

**Authors:** Atami De Main, Daphne Buitron, Irina Mindlis

**Affiliations:** Weill Cornell Medicine, New York, New York, United States; Weill Cornell Medicine, New York, New York, United States; Rutgers University, New York, New York, United States

## Abstract

The recruitment of Hispanic/Latino older adults living with multiple chronic conditions (MCC) for mental health research poses unique ethical and practical challenges that require careful consideration. This presentation aims to outline the strategies, facilitators, and barriers encountered while recruiting Hispanic/Latino older adults with MCC in senior centers across New York City for a mental health research initiative. Despite the increasing importance of involving this demographic in research, effective outreach and engagement remain challenging. We draw from our experience on a mixed-methods study designed to assess the underlying mechanisms influencing the relationship between MCC and mental health, while incorporating the perspectives of Hispanic/Latino older adults with MCC, to lay the groundwork for a community-based intervention to improve mental health in this population. Through our partnerships and collaborations with community-based organizations (CBOs), we were able to establish a shared understanding of our research goals and recruitment strategies. Key strategies to maximize participants’ engagement included using culturally relevant materials, via presentations, providing bilingual resources through flyers, and obtaining referrals from CBOs. Collaboration with site directors, staff engagement, as well as offering follow-up opportunities, significantly improved participation among Hispanic/Latino older adults in our study. However, we also identified several barriers, including site logistics, the method of survey delivery, and varying levels of survey literacy. These findings highlight the necessity for tailored recruitment approaches that address the unique needs and preferences of Hispanic/Latino older adults. Overall, this research contributes to the development of best practices for engaging underrepresented populations with MCC in mental health research.

